# A family of multi-spin rare-earth complexes based on a triazole nitronyl nitroxide radical: synthesis, structure and magnetic properties†

**DOI:** 10.1039/c8ra02546k

**Published:** 2018-04-24

**Authors:** Peng Yun Chen, Ming Ze Wu, Xiu Juan Shi, Li Tian

**Affiliations:** Tianjin Key Laboratory of Structure and Performance for Functional Molecules, Key Laboratory of Inorganic-Organic Hybrid Functional Materials Chemistry, Ministry of Education, Tianjin Normal University Tianjin 300387 P. R. China lilytianli@hotmail.com

## Abstract

The combination of Ln^III^ ions (Gd^III^, Tb^III^ or Dy^III^) and a triazole nitronyl nitroxide radical (4-Me-3-NITtrz) as spin carriers results in two mononuclear and one binuclear compounds, namely, [Ln(hfac)_3_(4-Me-3-NITtrz)(H_2_O)] (Ln = Gd(1), Tb(2); hfac = hexafluoroacetylacetone; 4-Me-3-NITtrz = 2-[3-(4-methyl-l,2,4-triazolyl)]-4,4,5,5-tetramethylimidazoline-1-oxyl-3-oxide) and [Dy(hfac)_2_(4-Me-3-NITtrz)_2_][Dy(hfac)_4_]·CHCl_3_. Compounds 1 and 2 are isostructural and crystallize in the *P*1̄ space group, whereas compound 3 crystallizes in the *P*2_1_/*n* space group. In 1 and 2, the central Ln^III^ ions are nine-coordinated (LnNO_8_) in a distorted spherical capped square antiprism geometry (*C*_4v_) finished by three bischelate hfac anions and one bidentate triazole radical and one aqua molecule. While 3 is a mixed-coordinated binuclear compound with Dy1 in a triangular dodecahedron (*D*_2d_) coordination sphere and Dy2 in a biaugmented trigonal prism (*C*_2v_) coordination sphere. Magnetic studies show that compound 2 exhibits field-induced single-molecule magnet (SMM) behavior.

## Abstract 2

Three Ln^III^–triazole–radical complexes have been synthesized, which exhibit interesting magnetic properties. The Tb^III^ complex shows frequency-dependent ac magnetic susceptibilities, which suggests the presence of slow magnetic relaxation.

## Graphical abstract

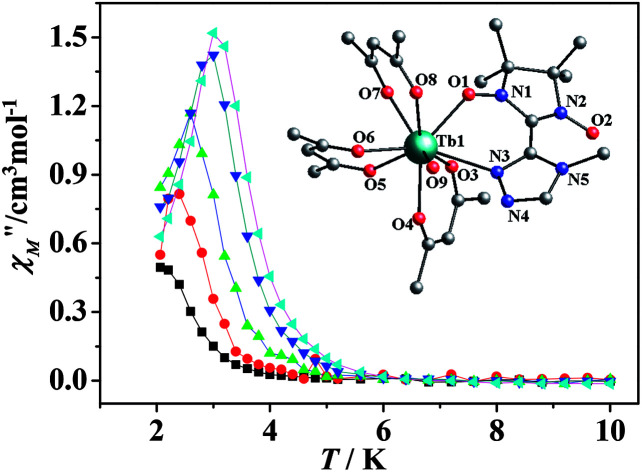

## Introduction

Low-dimensional molecular assemblies based on anisotropic metal ions that show slow relaxation of magnetization have attracted much attention.^1,2^ Such materials, named single-molecule magnets (SMMs) and single-chain magnets (SCMs), have latent applications in high-density data storage materials,^3^ quantum computations,^4^ and spintronic devices.^5^ For SMMs, reversal of magnetization arises from the combination of a large ground-state spin *S* and an Ising-type anisotropy. Recent studies show that rare-earth elements, especially heavy lanthanide ions such as terbium(iii) and dysprosium(iii), bearing huge magnetic moments and large intrinsic magnetic anisotropy, have become good candidates for the construction of SMMs.^6^ This has obviously increased the thermal energy barriers for the reversal of magnetization.^6*a*,7–11^ For example, the highest relaxation energy barrier 1837 K (twenty times higher than that in Mn_6_ (ref. 12)) was observed in a Dy^III^ SMM.^11^ However, the naturally accompanying quantum tunneling effect (QTM) resulting from the hyperfine couplings and dipolar spin–spin interactions of 4f ions always lowers the relaxation energy barrier and induces the loss of remnant magnetization,^13^ which is the inherent drawback for 4f-SMMs which can be observed even at liquid helium temperature. Among the various chemical routes investigated to obtain and improve the characteristics of 4f-SMMs, a strategy involving lanthanide ions with organic radicals has proved very successful.^13–16^ Rare-earth-radical based SMMs are appealing candidates owing to the Ising anisotropy of their metallic centers and the strong magnetic interactions between the radical and the metal ion, which may effectively quench the QTM and prevent the loss of magnetization. In particular, the N_2_^3−^ radical-bridged Tb^III^ complex exhibits hysteresis with a record blocking temperature up to 20 K.^16*d*^

**Scheme 1:**
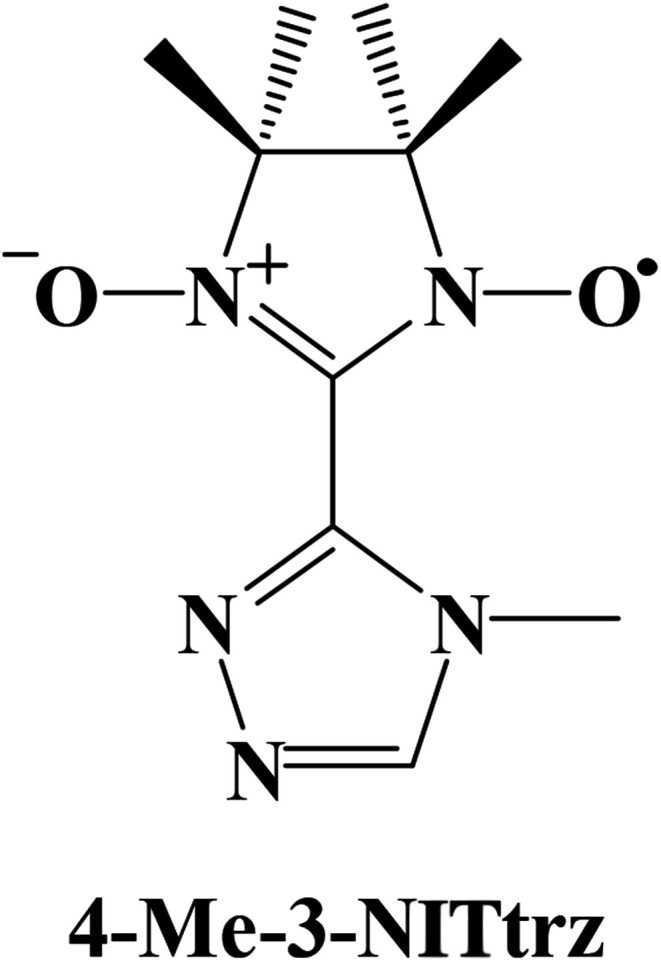
The structure of 4-Me-3-NITtrz.

Triazole ligands play a crucial role in the field of spin transition, and nitronyl nitroxide radicals are important in the fields of molecular magnets and nonlinear optics. Up to now, only four triazole nitronyl nitroxide radicals have been reported.^17^ All of the free radicals exhibit ferromagnetic interactions at low temperature, which proves that the π system and the N atoms in the triazole ring are good units for transferring effective magnetic interactions. Now we are interested in a nitronyl nitroxide radical based on a triazole ring, named 2-[3-(4-methyl-l,2,4-triazolyl)]-4,4,5,5-tetramethylimidazoline-1-oxyl-3-oxide (4-Me-3-NITtrz) (Scheme 1). So far, no rare-earth complexes with this radical have been reported. Herein we synthesize three lanthanide compounds based on radical 4-Me-3-NITtrz, namely, [Ln(hfac)_3_(4-Me-3-NITtrz)(H_2_O)] (Ln = Gd(1) and Tb(2)) and [Dy_2_(hfac)_6_(H_2_O)_2_]·1/2CH_2_Cl_2_ (3). Magnetic studies showed that complex 2 exhibits temperature-dependent ac susceptibility at low temperature, which suggests SMM behavior.

## Experimental details

### Materials and physical measurements

All of the reagents used in the syntheses were of analytical grade, except *n*-heptane and dichloromethane which were distilled after drying with Na and CaH_2_, respectively. Ln(hfac)_3_·2H_2_O was synthesized according to the method in the literature.^14*d*^ The radical 4-Me-3-NITtrz was prepared based on the procedure in the literature.^17*a*^ Elemental analyses for carbon, hydrogen, and nitrogen were performed on a PerkinElmer 240 elemental analyzer. Infrared spectra were recorded from KBr pellets in the 4000–400 cm^−1^ region on a Bruker TENOR 27 spectrometer. Powder X-ray diffraction measurements were recorded on a D/Max-2500 X-ray diffractometer using Cu-Kα radiation. Direct-current (dc) magnetic susceptibilities of crystalline samples were measured on an MPMS-7 SQUID magnetometer in the temperature range of 2–300 K with a 1000 Oe applied magnetic field. Diamagnetic corrections were made with Pascal's constants for all the constituent atoms and sample holders. Alternating-current (ac) susceptibilities were performed on the same magnetometer under a 0 or 3000 Oe dc field with an oscillation of 3.5 Oe.

### X-ray crystallography

Diffraction intensities were collected by using the *φ*–*ω* scan technique at 113 (for 1 and 2) or 157 K (for 3) on an Agilent SuperNova (Dual, Cu at zero, AtlasS2, CCD) diffractometer equipped with mirror-monochromated Cu-Kα radiation (*λ* = 1.54184 Å). Semiempirical multiscan absorption corrections were applied by SCALE3 ABSPACK, and the program CrysAlisPro^18^ was used for integration of the diffraction profiles. The structures were solved by direct methods and refined with the full-matrix least-squares technique using the ShelXT and ShelXL programs.^19^ Anisotropic thermal parameters were assigned to all non-H atoms. Some restraints, such as ISOR, DFIX, EADP, SADI, were applied to restrain the fluorine atoms and carbon atoms so as to avoid ADP problems on them. The organic hydrogen atoms were geometrically generated. H atoms attached to water molecules were located from difference maps and refined with isotropic temperature factors. Crystallographic data for the two compounds are listed in Table 1. Selected bond lengths of 1–3 are listed in Tables S1–S3.† CCDC 1831067, 1831068, and 1831069 contain the supplementary crystallographic data for compounds 1–3, respectively.

**Table 1:** Crystallographic data and structure refinement details for 1–3

	1	2	3
Formula	C_25_H_21_F_18_GdN_5_O_9_	C_25_H_21_F_18_N_5_O_9_Tb	C_51_H_39_Cl_3_Dy_2_F_36_N_10_O_16_
Mr	1034.72	1036.39	2163.27
Crystal system	Triclinic	Triclinic	Monoclinic
Space group	*P*1̄	*P*1̄	*P*2_1_/*n*
*a* (Å)	10.0094(3)	9.9958(3)	17.4350(4)
*b* (Å)	12.3115(4)	12.3076(4)	21.3325(7)
*c* (Å)	16.2016(5)	16.1878(6)	23.0646(5)
*α* (°)	105.182(3)	105.052(3)	90
*β* (°)	103.964(3)	103.900(3)	97.183(2)
*γ* (°)	99.368(3)	99.476(3)	90
*V* (Å^3^)	1814.66(10)	1811.3(2)	8511.2(4)
*Z*	2	2	4
*ρ*_calc_ (Mg m^−3^)	1.894	1.900	1.688
*μ* (mm^−1^)	1.975	10.990	11.432
*F* (000)	1008	1010	4200
*θ* range(°)	3.364–25.09	3.833–67.07	3.649–74.261
GOF on *F*^2^	1.030	1.042	1.060
*R*_1_/*wR*_2_ [*I* > 2*σ*(*I*)]	0.0394, 0.0868	0.0470, 0.1160	0.0739, 0.1933
*R*_1_/*wR*_2_ (all data)	0.0462, 0.0913	0.0522, 0.1205	0.0897, 0.2060

### Preparation of complexes of 1–3

Complexes 1 and 2 were obtained by the same procedure. A solution of Ln(hfac)_3_·2H_2_O (0.1 mmol) in dry heptane (15 mL) remained refluxing for 3 h and then cooled down to 60 °C, to which 4-Me-3-NITtrz (24 mg, 0.1 mmol) in CH_2_Cl_2_ (5 mL) was added. The resulting solution was stirred with refluxing for 1 h and then cooled to room temperature. After filtration, the final solution was stored in a refrigerator at 0–4 °C for about ten days to give blue-violet crystals, which were suitable for X-ray analysis.

#### [Gd(hfac)_3_(4-Me-3-NITtrz)(H_2_O)] (1)

Yield 0.050 g, 48%. Elemental analysis calcd (%) for 1 (C_25_H_21_F_18_GdN_5_O_9_: 1034.72): C, 29.02; H, 2.04; N, 6.77. Found: C, 28.74; H, 1.98; N, 6.61.

#### [Tb(hfac)_3_(4-Me-3-NITtrz)(H_2_O)] (2)

Yield 0.054 g, 52%. Elemental analysis calcd (%) for 2 (C_25_H_21_F_18_N_5_O_9_Tb: 1036.39): C, 28.97; H, 2.04; N, 6.76. Found: C, 28.68; H, 2.14; N, 6.53.

Complex 3 was obtained by the following method. A suspension of 0.1 mmol Dy(hfac)_3_·2H_2_O in dry heptane (10 mL) remained refluxing for 1 h and then cooled to 75 °C, to which 4-Me-3-NITtrz (24 mg, 0.1 mmol) in CHCl_3_ (5 mL) was added. The mixture was stirred with refluxing for 0.5 h and then cooled to room temperature. After filtration, slow evaporation of the final solution for about two weeks in a refrigerator at 0–4 °C gave violet strip crystals suitable for single-crystal X-ray analysis.

#### [Dy(hfac)_2_(4-Me-3-NITtrz)_2_][Dy(hfac)_4_]·CHCl_3_ (3)

Yield 0.032 g, 23%. Elemental analysis calcd (%) for 3 (C_51_H_39_Cl_3_Dy_2_F_36_N_10_O_16_: 2163.27): C, 28.31; H, 1.82; N, 6.48. Found: C, 28.22; H, 1.92; N, 6.26.

## Results and discussion

### Crystal structure

#### Structure of 1 and 2

**Fig. 1:**
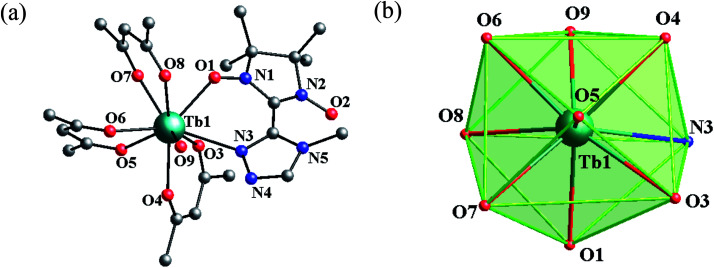
(a) Simplified view of the crystal structures of 2. Fluorine, hydrogen, and some carbon atoms are omitted for clarity. (b) Polyhedral configuration of the terbium atom in complex 2.

Single-crystal X-ray diffraction indicates that complexes 1 and 2 are isomorphous and belong to the triclinic *P*1̄ space group with *Z* = 2. Complex 2 is regarded as representative to describe the crystal structure. In 2, the asymmetric unit contains one crystallographically independent molecule with the central terbium ion in the TbNO_8_ coordination sphere. As shown in Fig. 1, Tb1 is surrounded by three bischelate hfac anions, one 4-Me-3-NITtrz radical ligand and one aqua molecule with a slightly distorted capped square antiprism (*C*_4v_) polyhedron configuration. Each MeTrzNIT acts as a bidentate chelate ligand and is coordinated to the same Tb^III^ ions through one NO group and one nitrogen atom of the triazole ring. The Tb–O bond length associated with the NO group is 2.357(2) Å with the corresponding Tb–O–N angle of 130.3(3)°. The Tb–N distance (2.698(4) Å) is a little longer than normal Tb–N bonds. Other selected bond lengths and angles are listed in the ESI (Table S2†). When applying the *C*_4v_ symmetry to the TbNO_8_ site, the CSM method gives the minimal derivation value from the ideal model with *S* = 0.394. The neighboring molecules are connected by weak hydrogen-bonding C–H⋯F interactions into a 3D supermolecular network with the shortest O–O (NO) contact of 9.996 Å (O2⋯O2#1) (Fig. S3 and S4, ESI†).

#### Structure of 3

**Fig. 2:**
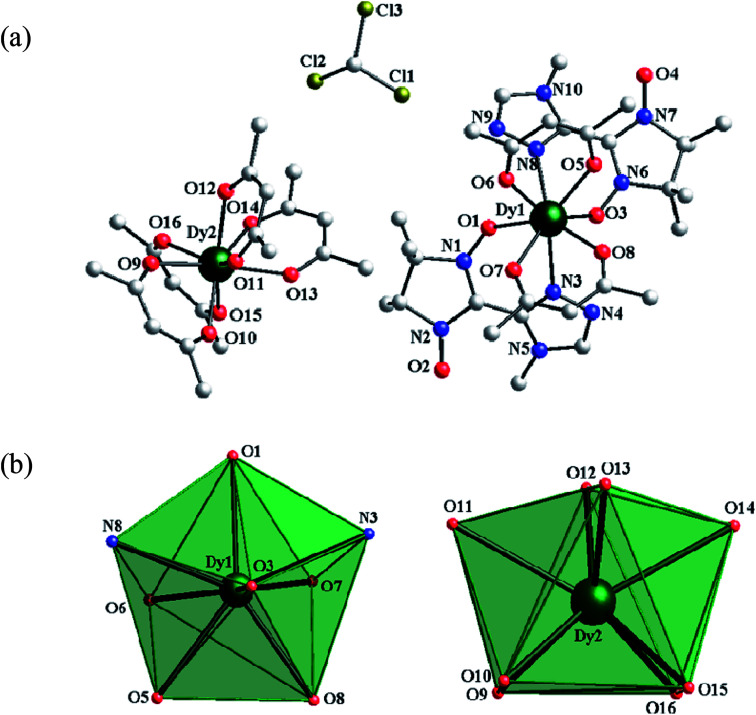
(a) The asymmetric coordination unit in complex 3. Fluorine, hydrogen, and some carbon atoms are omitted for clarity. (b) Polyhedral configurations of Dy1 and Dy2 in complex 3.

Compound 3 belongs to the monoclinic *P*2_1_/*n* space group and consists of one mononuclear [Dy(hfac)_2_(4-Me-3-NITtrz)_2_] anion, one [Dy(hfac)_4_] counter cation and a free chloroform. The asymmetric unit and the polyhedral of the Dy^III^ ions are shown in Fig. 2. Dy1 is in the DyN_2_O_6_ coordinating environment completed by four oxygen atoms from two bischelate hfac, one two bidentate 4-Me-3-NITtrz radical with the NO group and a triazole nitrogen atom. Dy2 is surrounded by eight oxygen atoms from four hfac anions. The Dy–O(hfac) distances range from 2.304(5) to 2.400(5) Å, and the two Dy–O_rad_ bond lengths are 2.307(5) and 2.311(5) Å, respectively. Dy1 is estimated as a triangular dodecahedron (*D*_2d_) coordination sphere with the deviation parameter *S* = 1.223, while Dy2 is in a biaugmented trigonal prism (*C*_2v_) coordination sphere with *S* = 0.932.

#### Static magnetic properties

**Fig. 3:**
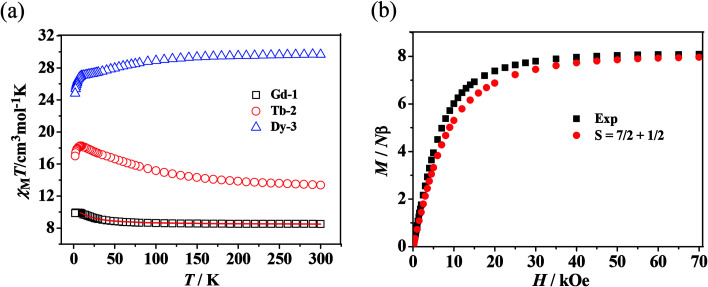
(a) *χ*_M_*T vs. T* plots for 1–3 where the solid lines represent the theoretical values based on the corresponding equations. (b) Field dependence of the magnetization at 2 K for complex 1.

The temperature-dependent magnetic susceptibilities of complexes 1–3 were performed under 1 kOe in the temperature range 2–300 K. The phase purity of the bulk samples was confirmed by XRD analyses and the experimental and simulated curves are shown in Fig. S5 and S6 (ESI).† The magnetic behaviors of the three complexes were studied and are shown in Fig. 3.

For 1, the value of *χ*_M_*T* at 300 K is 8.52 cm^3^ K mol^−1^, a little higher than the theoretical value of 8.15 cm^3^ K mol^−1^ for an uncoupled Gd^III^ ion (^8^*S*_7/2_, *g* = 2) plus an organic radical (*S* = 1/2, *g* = 2). Following a decrease in temperature, the value of *χ*_M_*T* increases slightly up to 55 K, and then shows an abrupt increase to 9.94 cm^3^ K mol^−1^ at 2.5 K. The overall magnetic behavior indicates the presence of ferromagnetic coupling between the Gd^III^ ion and the nitroxide radical.

The system was regarded as a bi-spin unit, and the magnetic simulation was carried out by using the spin Hamiltonian: *H* = −2*J*_Gd–Rad_*Ŝ*_Gd_*Ŝ*_Rad_, in which *J*_Gd–Rad_ represented the exchange coupling of the Gd^III^–radical. The mean-field approximation (*zj*′) was introduced to indicate the possible intermolecular interactions. The magnetic data were analyzed with the following equations.1
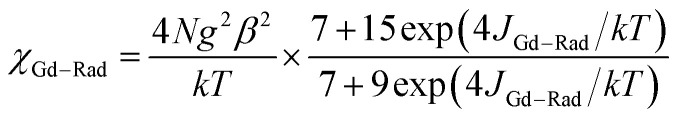
2
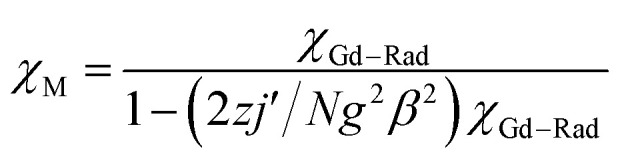


The experimental curve is better reproduced (Fig. 3) by the approximate eqn (1) and (2), giving fitting parameters of *g* = 2.02, *J*_Rad–Gd_ = 4.35 cm^−1^, and *zj*′ = −0.006 cm^−1^. The positive value of *J*_Gd–Rad_ reveals the presence of ferromagnetic interaction between Gd(iii) and the radical, which is very common in Gd(iii)–radical systems.^1*a*,20^ This ferromagnetic interaction is also corrected by the magnetization measurement at 2 K, where the *M*(*H*) curve remains above the Brillouin curve for an isolated spin of *S* = 1/2 + 7/2 (Fig. 3(b)). A magnetization of 8.01 *Nβ* is reached at 50 kOe, in agreement with the 7.98 *Nβ* for the ferromagnetic arrangement of the bi-spins.

As shown in Fig. 3, the observed room-temperature *χ*_M_*T* value for complex 2 is 12.31 cm^3^ K mol^−1^, which is very close to the expected value of 12.10 cm^3^ K mol^−1^ for one free Tb(iii) ion and one radical. Upon cooling, the *χ*_M_*T* value continuously increased until it reached a maximum of 18.22 cm^3^ K mol^−1^ at 9 K, and then decreased to 16.97 cm^3^ K mol^−1^ at 2.0 K. The overall behavior indicates that there exists a ferromagnetic interaction between the Tb(iii) ion and the radical, which is observed in other Tb–radical compounds.^20*b*,21^ The decrease in *χ*_M_*T* below 9 K may be attributed to intermolecular magnetic coupling. For 3, the room temperature value of *χ*_M_*T* is 29.66 cm^3^ K mol^−1^, a little higher than the theoretical value of 29.09 cm^3^ K mol^−1^ for two free Dy^III^ ions plus two isolated radicals. As the temperature is lowered, the *χ*_M_*T* value decreases slightly until 10 K, when it begins to decrease quickly as the temperature is lowered further and reaches the lowest value of 24.82 cm^3^ K mol^−1^ at 2 K. In the high-temperature range, the decrease in *χ*_M_*T* value is ascribed to the depopulation of the Ln^III^ stark sublevels and/or the Ln–radical interactions. In the low-temperature range, the decrease in *χ*_M_*T* value can be attributed to the antiferromagnetic Ln^III^–radical interaction.

For both the complexes, the field-dependent magnetization value shows a rapid increase at low fields (Fig. S7 and 8, ESI†). For 2, *M* increases up to 7.17 *Nβ* at 70 kOe, which is much lower than the saturation value of 10 *Nβ* (9 *Nβ* for each Tb^III^ ion for *J* = 6 and *g* = 3/2, plus 1 *Nβ* for one organic radical) (Fig. S7, see ESI†). For 3, the magnetization increases up to 15.09 *Nβ* at 70 kOe with an increase in the applied field, which also does not reach the expected saturation value of 22 N (Fig. S8, see ESI†). Taking into account the strong spin–orbit coupling in Ln^III^ ions, the large gaps between experimental data and theoretical saturation values for compounds 2 and 3 can be attributed to the magnetic anisotropy and/or low-lying excited states in the systems.^22^

#### Dynamic magnetic properties

**Fig. 4:**
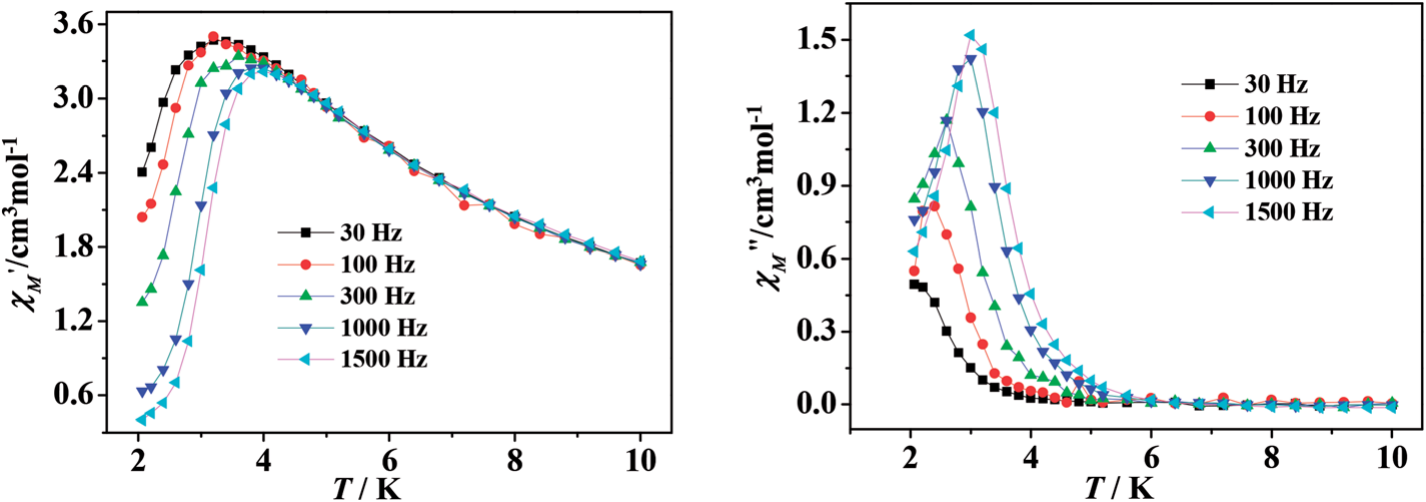
Temperature dependence of the real (left) and imaginary (right) components of ac magnetic susceptibility for 2 under a 3000 Oe dc field.

**Fig. 5:**
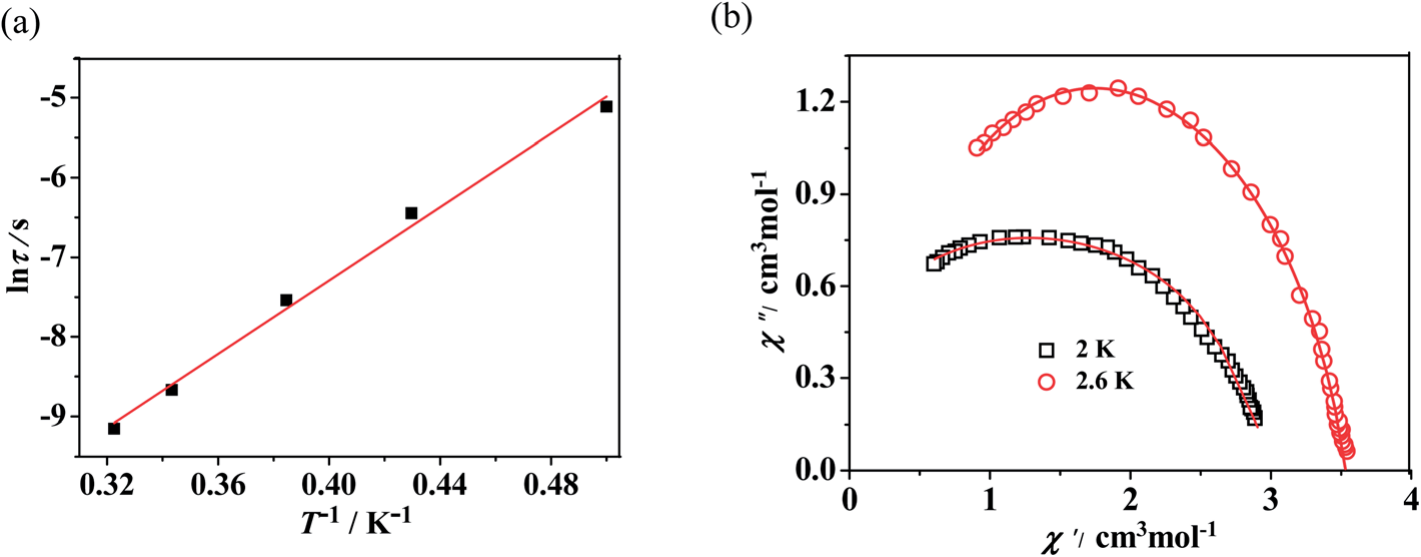
(a) Magnetization relaxation time, ln *τ vs T*^−1^ plot for 2 under 3000 Oe dc field. The red line is the fitting result based on the Arrhenius law. (b) Cole–Cole plots measured at 2 K and 2.6 K under 3000 Oe for 2; the solid lines are the best fit to the experimental data by using the Debye model.

In order to examine the spin dynamics of compounds 2 and 3, alternating current (ac) measurements were carried out under a zero dc field or 3000 Oe with an oscillation of 3.5 Oe. For the mononuclear complex 2, temperature-dependent ac signals are observed in both in-phase and out-of-phase components with no peaks observed, which suggests that a very fast magnetization relaxation process may exist (Fig. S9, see ESI†). It is generally known that magnetization can reverse *via* a quantum mechanical tunneling process in lanthanide SMMs within the lowest energy doublet. However, the application of a static magnetic field can suppress QTM significantly. Here a dc field of 3000 Oe is used to probe the dynamic behavior of ac magnetic susceptibility (Fig. 4). Accordingly, well-resolved peaks emerge, which mean that the relaxation process is slowed by the external field. The relaxation time *τ* is extracted from the maximums of the imaginary component of the ac susceptibility based on the temperature-dependent data. The plot of ln *τ versus T*^−1^ displays linear dependence, indicating that the relaxation follows a thermally activated Orbach mechanism (Fig. 5(a)). An effective energy gap of 16 K and a pre-exponential *τ*_0_ of 6.7 × 10^−8^ s were simulated from the Arrhenius law (*τ* = *τ*_0_ exp(*Δ*_eff_/*k*_B_*T*)), which fall within the range of SMMs.^13–16^ As shown in Fig. 5(b), Cole–Cole plots exhibit nearly semicircular shapes. The generalized Debye model is used to extract the distribution parameters *α* and gives values of 0.32 (2 K) and 0.23 (2.6 K), indicating a single relaxation process. For complex 3, no apparent out-of-phase signals are observed above 2 K, which indicates that no slow relaxation exists (Fig. S11, see ESI†).

For the lanthanide-containing complexes, the crystal field created by the surrounding ligands dramatically influenced the local anisotropy of the Ln^III^ ions, which is crucial to the slow relaxation of magnetization in SMMs. As in this work, the Tb^III^ compound exhibits field-induced SMM behavior, while the Dy^III^ compound does not show any obvious signals in the out-of-phase ac magnetic susceptibility. Considering the Kramers nature of Dy^III^ ions, the double degeneracy of the ground state, ±*m*_J_, is always ensured in a zero applied dc field.^23^ The absence of slow relaxation of magnetization might result mostly from the small energy gap between the ground state and the first excited state in a certain local symmetry. Whereas for the non-Kramers Tb^III^ ion, the spin-coupled ground state *J* = 6 can be split by the axial symmetry crystal field, leading to the lowest substate of *m*_J_ = ±6 having a large gap (more than 400 cm^−1^) from the second-lowest substate. However, the low axial symmetry of *C*_4v_ geometry may remove the double degeneracy to some extent and reduce the energy barrier required for spin reversal. In addition, the quantum tunneling inducted by a transverse field and/or dipole–dipole interactions also decreases the final *U*_eff_ observed.

## Conclusions

In conclusion, a triazole nitronyl nitroxide radical (4-Me-3-NITtrz) and three new compounds [Ln(hfac)_3_(4-Me-3-NITtrz)(H_2_O)] (Ln = Gd (1), Tb(2)) and [Dy(hfac)_2_(4-Me-3-NITtrz)_2_][Dy(hfac)_4_]·CHCl_3_ have been synthesized. For complex 1, the fitted results from the magnetic susceptibility reveal the presence of ferromagnetic interactions between Gd(iii) and the radical, with *J*_Rad–Gd_ = 4.35 cm^−1^. The Tb^III^ compound exhibits field-induced SMM behavior with a *U*_eff_ of 16 K and *τ*_0_ of 6.7 × 10^−8^ s under a 3000 Oe external field, while the Dy^III^ compound shows no obvious out-of-phase ac signal. The results demonstrated that the local symmetry of central lanthanide ions is an important factor for magnetic anisotropy and for the rational design of new lanthanide–radical SMMs.

## Conflicts of interest

There are no conflicts to declare.

## Supplementary Material

RA-008-C8RA02546K-s001

RA-008-C8RA02546K-s002
