# Supplementary material for: A family of multi-spin rare-earth complexes based on a triazole nitronyl nitroxide radical: synthesis, structure and magnetic properties
Source: RSC Adv. 2018 Apr 24;8(28):15480–6. doi: 10.1039/c8ra02546k (PMC9080062; doi:10.1039/c8ra02546k)
Supplement: RA-008-C8RA02546K-s001 [file RA-008-C8RA02546K-s001.pdf]

Electronic Supplementary Material (ESI) for RSC Advances.
This journal is © The Royal Society of Chemistry 2018

Supporting Information

A family of multi-spin rare-earth complexes based on a triazole nitronyl nitroxide radical: synthesis, structure and magnetic properties

Peng Yun Chen, Ming Ze Wu, Xiu Juan Shi, and Li Tian*

*Tianjin Key Laboratory of Structure and Performance for Functional Molecules, Key Laboratory of Inorganic-Organic Hybrid Functional Materials Chemistry, Ministry of Education, Tianjin Normal University, Tianjin 300387, P. R. China. *Email: lilytianli@hotmail.com*

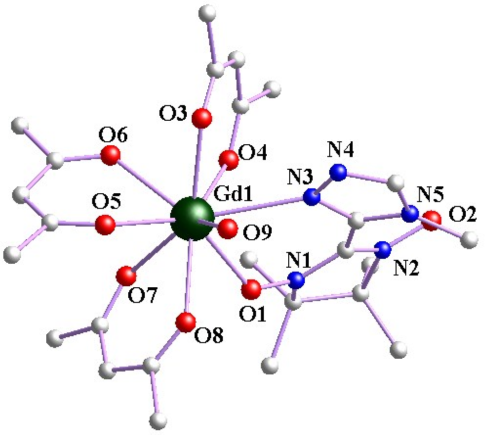

Fig. S1 Simplified view of the crystal structures of **1**.

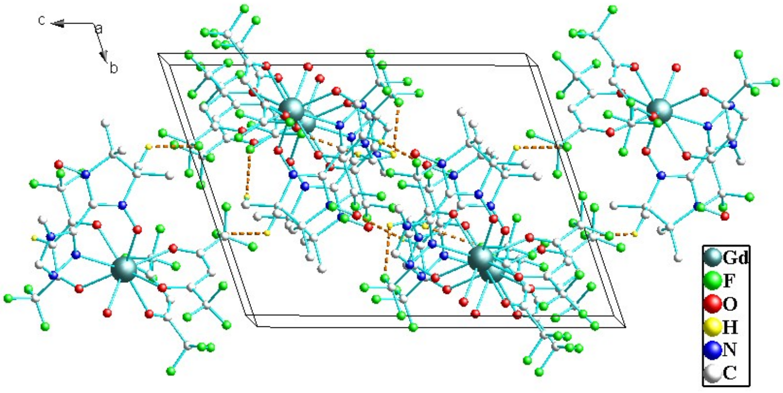

Fig. S2 Viewing of the 3D packing structure of **1**.

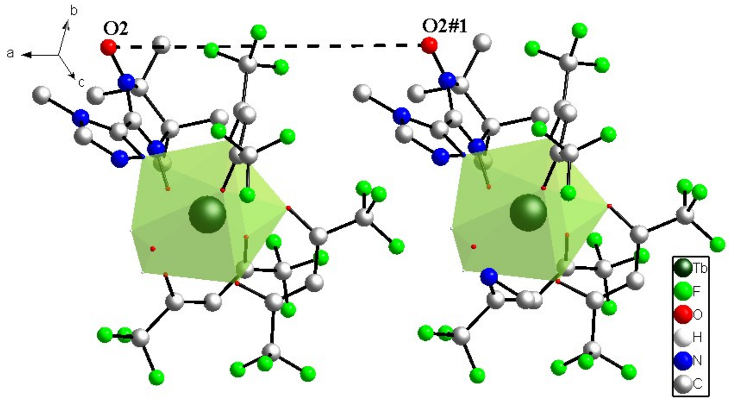

Fig. S3 The shortest O···O distance in **2**. (O2···O2#1: 9.996 Å).

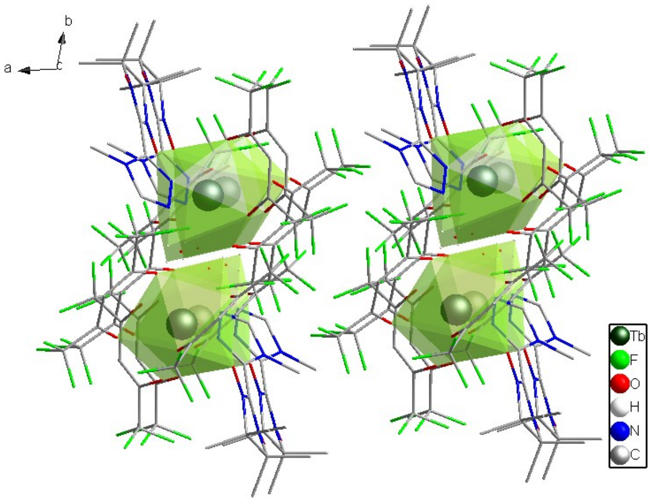

Fig. S4 Viewing of the 3D packing structure of **2**.

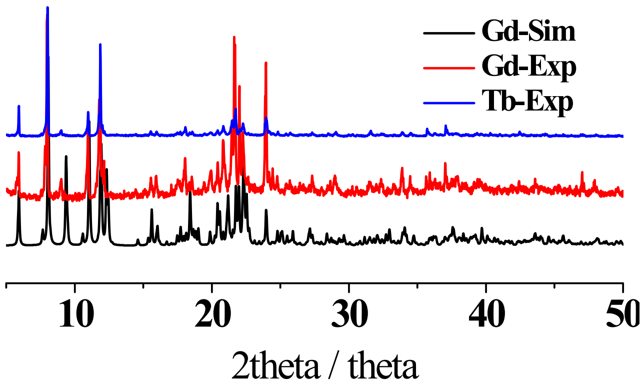

Fig. S5 Powder X-ray diffractions of **1-2**.

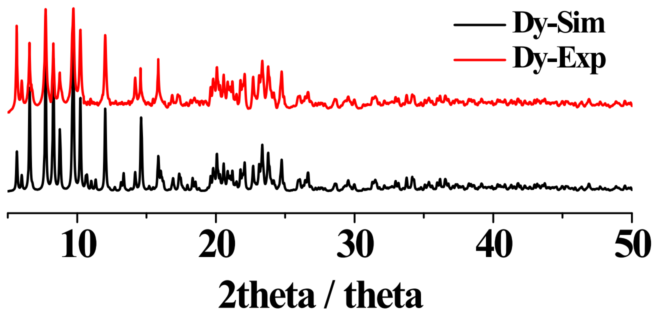

Fig. S6 Powder X-ray diffractions of **3**.

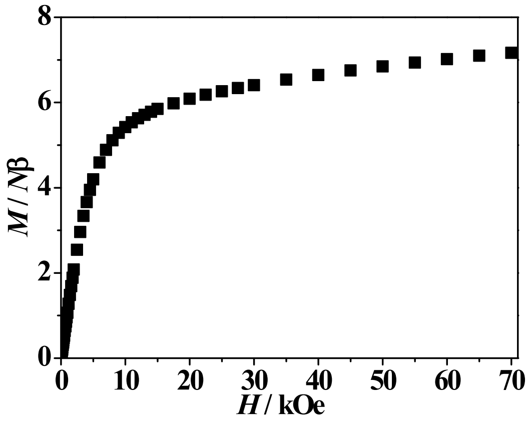

Fig. S7 Field dependence of the magnetization at 2 K for complex **2**.

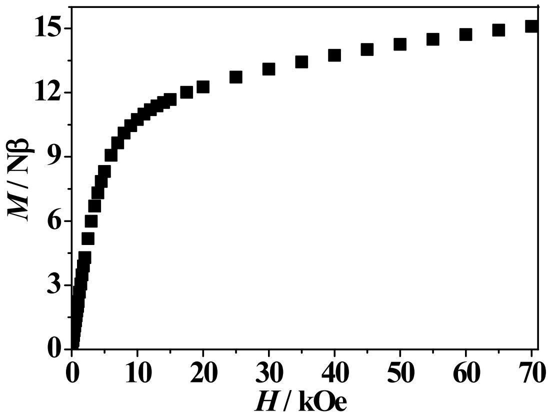

Fig. S8 Field dependence of the magnetization at 2 K for complex **3**.

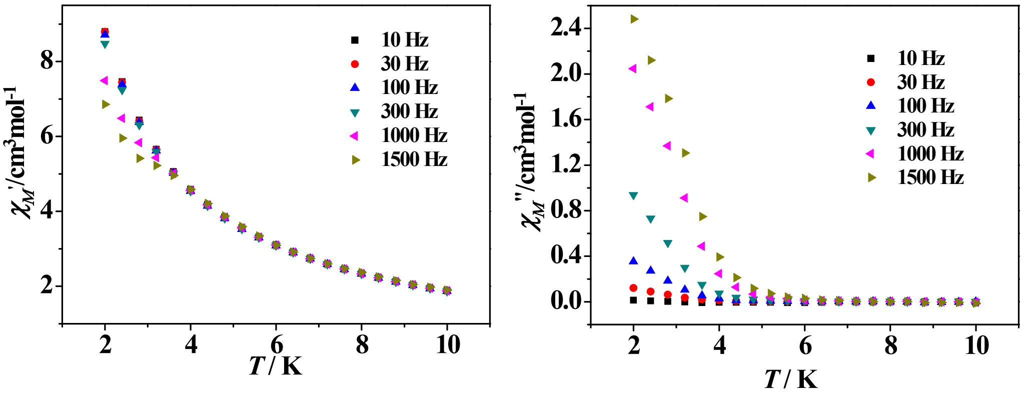

Fig. S9 Temperature dependence of the in-phase (χ') (left) and out-of-phase (χ'') (right) components of the ac magnetic susceptibility for complex **2** under zero dc field.

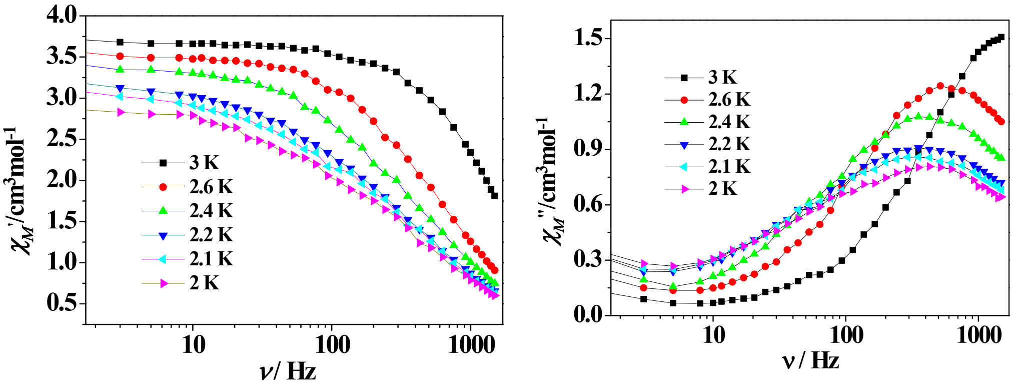

Fig. S10 Frequency dependence of the in-phase (χ') and out-of-phase (χ'') components of the ac magnetic susceptibility for complex **2** under 3000 Oe dc field.

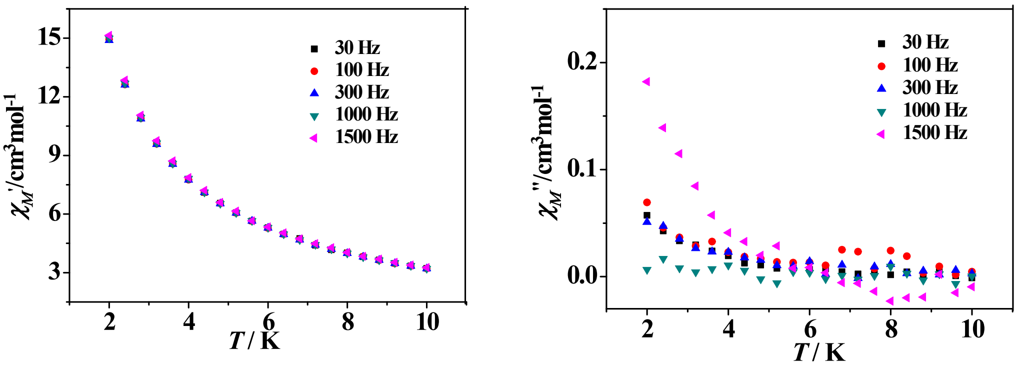

Fig. S11 Temperature dependence of the in-phase (χ') (left) and out-of-phase (χ'') (right) components of the ac magnetic susceptibility for complex **3** under zero dc field.

Table S1. Selected bond lengths (Å) and bond angles (°) in complex **1**.

Gd(1)-O(1)	2.423(3)	O(4)-Gd(1)-O(1)	75.61(11)
Gd(1)-O(3)	2.380(3)	O(4)-Gd(1)-O(5)	138.98(12)
Gd(1)-O(4)	2.382(3)	O(4)-Gd(1)-O(9)	131.31(11)
Gd(1)-O(5)	2.388(3)	O(4)-Gd(1)-N(3)	67.54(12)
Gd(1)-O(6)	2.468(4)	O(5)-Gd(1)-N(3)	140.10(11)
Gd(1)-O(7)	2.375(3)	O(5)-Gd(1)-O(1)	135.81(11)
Gd(1)-O(8)	2.465(3)	O(5)-Gd(1)-O(6)	69.73(11)
Gd(1)-O(9)	2.397(3)	O(6)-Gd(1)-N(3)	129.94(11)
Gd(1)-N(3)	2.701(4)	O(7)-Gd(1)-O(1)	71.02(11)
O(1)-Gd(1)-O(6)	124.36(11)	O(7)-Gd(1)-O(4)	93.17(11)
O(1)-Gd(1)-O(8)	68.37(10)	O(7)-Gd(1)-N(3)	137.52(11)
O(1)-Gd(1)-N(3)	67.68(11)	O(8)-Gd(1)-O(6)	128.44(11)
O(3)-Gd(1)-O(1)	138.02(10)	O(8)-Gd(1)-N(3)	101.56(12)
O(3)-Gd(1)-O(4)	74.60(11)	O(9)-Gd(1)-O(1)	105.75(11)
O(3)-Gd(1)-N(3)	73.91(11)	O(9)-Gd(1)-N(3)	68.52(11)

Table S2. Selected bond lengths (Å) and bond angles (°) in complex **2**.

Tb(1)-O(1)	2.415(4)	O(4)-Tb(1)-O(1)	138.10(11)
Tb(1)-O(3)	2.367(3)	O(4)-Tb(1)-O(5)	70.43(11)
Tb(1)-O(4)	2.367(3)	O(4)-Tb(1)-O(9)	73.79(12)
Tb(1)-O(5)	2.465(4)	O(4)-Tb(1)-N(3)	73.58(12)
Tb(1)-O(6)	2.364(3)	O(5)-Tb(1)-N(3)	129.51(11)
Tb(1)-O(7)	2.366(3)	O(6)-Tb(1)-O(1)	135.69(11)
Tb(1)-O(8)	2.449(3)	O(6)-Tb(1)-O(4)	85.31(11)
Tb(1)-O(9)	2.397(3)	O(6)-Tb(1)-N(3)	139.96(12)

Tb(1)-N(3)	2.698(4)	O(7)-Tb(1)-O(1)	70.92(11)
O(1)-Tb(1)-O(5)	124.32(12)	O(7)-Tb(1)-O(4)	139.20(12)
O(1)-Tb(1)-O(8)	68.37(11)	O(7)-Tb(1)-N(3)	137.60(12)
O(1)-Tb(1)-N(3)	67.97(11)	O(8)-Tb(1)-O(5)	128.60(10)
O(3)-Tb(1)-O(1)	75.36(11)	O(8)-Tb(1)-N(3)	101.85(11)
O(3)-Tb(1)-O(8)	143.43(11)	O(9)-Tb(1)-O(5)	105.94(12)
O(3)-Tb(1)-N(3)	67.43(12)	O(9)-Tb(1)-N(3)	68.68(11)

Table S3. Selected bond lengths (Å) and bond angles (°) in complex **3**.

Dy(1)-O(1)	2.307(5)	O(3)-Dy(1)-O(5)	83.06(19)
Dy(1)-O(3)	2.311(5)	O(3)-Dy(1)-O(6)	145.91(17)
Dy(1)-O(5)	2.347(5)	O(3)-Dy(1)-N(3)	69.67(17)
Dy(1)-O(6)	2.311(5)	O(5)-Dy(1)-N(3)	140.19(17)
Dy(1)-O(7)	2.334(5)	O(5)-Dy(1)-N(8)	70.48(18)
Dy(1)-O(8)	2.304(5)	O(6)-Dy(1)-O(5)	74.02(18)
Dy(1)-N(3)	2.537(6)	O(6)-Dy(1)-O(7)	74.56(18)
Dy(1)-N(8)	2.527(6)	O(6)-Dy(1)-N(3)	71.22(18)
Dy(2)-O(9)	2.325(4)	O(7)-Dy(1)-O(5)	123.1(2)
Dy(2)-O(10)	2.335(4)	O(7)-Dy(1)-N(3)	139.20(12)
Dy(2)-O(11)	2.399(6)	O(8)-Dy(1)-O(3)	88.40(19)
Dy(2)-O(12)	2.341(5)	O(8)-Dy(1)-O(5)	71.98(19)
Dy(2)-O(13)	2.307(5)	O(8)-Dy(1)-N(3)	78.6(2)
Dy(2)-O(14)	2.400(5)	N(8)-Dy(1)-N(3)	122.0(2)
Dy(2)-O(15)	2.339(6)	O(9)-Dy(2)-O(10)	74.26(17)
Dy(2)-O(16)	2.326(5)	O(9)-Dy(2)-O(12)	87.61(16)
O(1)-Dy(1)-O(3)	93.42(19)	O(10)-Dy(2)-O(12)	141.61(18)
O(1)-Dy(1)-O(5)	141.67(18)	O(10)-Dy(2)-O(15)	73.65(19)
O(1)-Dy(1)-O(7)	83.19(19)	O(12)-Dy(2)-O(14)	69.39(18)
O(1)-Dy(1)-N(3)	70.59(19)	O(13)-Dy(2)-O(15)	86.2(2)
O(1)-Dy(1)-N(8)	70.48(18)	O(15)-Dy(2)-O(14)	75.85(19)
